# Fecal Recovery of Probiotics Administered as a Multi-Strain Formulation during Antibiotic Treatment

**DOI:** 10.3390/biomedicines8040083

**Published:** 2020-04-09

**Authors:** Sofia D. Forssten, Nicolas Yeung, Arthur C. Ouwehand

**Affiliations:** DuPont Nutrition and Biosciences, Sokeritehtaantie 20, 02460 Kantvik, Finland; sofia.forssten@dupont.com (S.D.F.); nicolas.yeung@dupont.com (N.Y.)

**Keywords:** probiotic, *Lactobacillus paracasei*, *Lactobacillus acidophilus*, *Bifidobacterium lactis*, AAD, Antibiotic associated diarrhea, Fecal recovery

## Abstract

The present study aimed to investigate whether probiotic recovery is affected when consumed together with antibiotics. Fecal samples were collected from an earlier antibiotic associated diarrhea, randomized, placebo-controlled study with a product consisting of a combination of *Lactobacillus acidophilus* NCFM, *Lactobacillus paracasei* Lpc-37, and *Bifidobacterium lactis* Bi-07, *B. lactis* Bl-04 at equal numbers and at a total dose of 10^10^ CFU. Fecal samples were collected during the screening visit (T0), i.e., at the time of antibiotic prescription, and then on the last day of the antibiotic treatment (T1) as well as seven days after the subject had stopped taking the antibiotic treatment (T2) and at two weeks after completing antibiotic treatment and one week after probiotic/placebo consumption stopped (T3). Samples were analyzed for the presence of the four administered strains. The study was registered at clinicaltrials.gov as NCT01596829. Detection levels of all four strains were significantly increased from T0 to T1 and returned to baseline level from T2 to T3. There were also significantly more subjects with detectable levels of *L. paracasei* Lpc-37, *B. lactis* Bi-07, and *B. lactis* Bl-04 at T1 and T2 compared to T0 and T3, and compared to placebo. Each of the four strains could be detected in the feces of patients apparently unaffected by the simultaneous consumption of antibiotics.

## 1. Introduction

Antibiotic-associated diarrhea (AAD) is one of the most frequently encountered adverse effects following antibiotic administration and is the leading cause of diarrhea in hospitalized patients [1]. It is a major public health concern and represents an important source of morbidity and mortality. The incidence of AAD, as reported in the literature, ranges between 5% and 39% [2,3] and varies according to individual susceptibility, the environment in which the patient resides, the regimen of antibiotic use (dose, duration) and the family of antibiotics administered. Risk factors include age (<6 years and >65 years), comorbidities, immunological status of the patient, the type and duration of antibiotic administered, along with the duration of hospitalization (2). All classes of antibiotics can produce AAD, but broad-spectrum antibiotics such as the extended spectrum penicillins, cephalosporins, and clindamycin have been shown to produce a higher rate of AAD [4]. Probiotics have been shown to be effective in reducing the risk for AAD and other side effects associated with antibiotic use [5]. In this study, out-patients with common infections requiring antibiotic treatment were recruited and randomized to receive a multi-strain probiotic formulation or placebo. The probiotic product has earlier been shown to reduce the incidence and duration of AAD [6]. The study aimed to recruit 400 patients but was terminated when 258 patients were enrolled due to the low incidence of AAD (1.1% in the probiotic group and 6.1% in the placebo group; *p* > 0.05); no *Clostridioides difficile*-associated diarrhea was observed. Although the primary aim of the study was thus not met, we here report on the fecal detection of the four strains that composed the probiotic formulation to determine if there was any interaction between the strains in vivo. In an earlier study, we have shown that there does not appear to be a negative interaction between the components of this multi-strain probiotic in a simulated colonic microbiota ecosystem [7]. However, an in vivo test on the potential interaction of strains in a multi-strain probiotic is still lacking. Furthermore, an often-returning question is whether the probiotics will survive when consumed together with antibiotics. The aim of this study was to answer these two questions.

## 2. Materials and Methods

### 2.1. Study Population

The clinical study was performed to investigate the effect of probiotic supplementation on reducing the risk for AAD, as well as to assess the response effect of probiotics on the fecal microbiota and on product safety. The study was a two-arm, parallel group, placebo-controlled, double-blind, randomized clinical trial, stratified by age (18–49 and 50–65; 50% each), gender (equal numbers male and female), and number of days on antibiotics (3–8 days or 9–14 days; 50% each). Out-patients with common infections were recruited if best practice antibiotic treatment for the diagnosed infection fell into the categories of broad spectrum penicillins (e.g., amoxycillin or ampicillin alone or in combination with beta-lactamase inhibitors); narrow spectrum penicillins (e.g., isoxazolylpenicillin, phenoxymethylpenicillin); cephalosporins; doxicyclin (or other tetracyclins); clarithromycin (or other macrolides, e.g., erythromycin and azithromycin); ciprofloxacin (or other fluoroquinolones, e.g., levofloxacin and norfloxacin), nitrofurantoin, trimethoprim, and sulfadiazine, as well as antibiotic treatment expected to be 3 to 14 days in duration. Patients started the consumption of the investigational product (i.e., probiotics or placebo) on the same day as the start of their antibiotic treatment and continued for 7 days with the consumption of the investigational product after the antibiotic treatment was completed [6]. Patients were instructed to consume antibiotics and probiotics at least 2 h apart.

The investigational product was a combination of probiotics *Lactobacillus acidophilus* NCFM (ATCC 700396), *Lactobacillus paracasei* Lpc-37 (ATCC SD5275), *Bifidobacterium animalis* subsp. *lactis* Bi-07 (ATCC SD5220), and *Bifidobacterium animalis* subsp. *lactis* Bl-04 (ATCC SD5219) at equal numbers and at a total dose of 10^10^ colony forming units (CFU) or placebo. The placebo consisted of microcrystalline cellulose, which was used as excipient in the probiotic product. Both products were provided in hydroxypropylmethyl cellulose capsules (size 0).

This human intervention study was conducted according to the guidelines laid down in the Declaration of Helsinki and all procedures involving human subjects/patients were approved (17 April 2011) by the Ethics Committee of Pirkanmaa Hospital District, Finland (ETL-code R12066), and registered at clinicaltrial.gov as NCT01596829. 

### 2.2. Sampling

**Figure 1:**
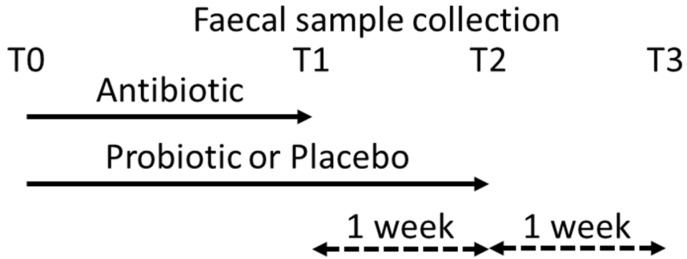
Schematic representation of fecal sample collection and study timeline. T0 is baseline, T1 is at the end of antibiotic treatment, T2 is one week after antibiotic treatment was stopped and the last day of probiotic supplementation, and T3 is two weeks after antibiotic treatment was stopped and one week after probiotic supplementation was stopped.

Fecal samples were collected from 96 subjects (50 receiving placebo and 46 receiving the probiotic) during the screening visit (T0) before the start of the intervention, i.e., at the time of antibiotic prescription, and then on the last day of the antibiotic treatment ±1 day (T1) as well as 7 days after the subject had stopped taking the antibiotic treatment (T2). A final fecal sample was collected two weeks after completing antibiotic treatment and one week after finishing the consumption of the investigational product (T3); Figure 1. Fecal samples were immediately frozen and stored at −18 °C, or lower, after they had been collected.

### 2.3. Extraction and Quantification of Bacterial DNA

The four probiotic strains included in the investigational product were obtained separately from the supplier (HOWARU^®^ Restore, Danisco Deutschland, Niebüll) as well as the commercial capsules with their exact probiotic composition. Strain specific qPCR assays were designed and optimized in order to detect and quantify each strain. Bacterial DNA from the pure single strains as well as DNA from the fecal sample of the subjects in the clinical trial was extracted and purified with an automated MagMAX™ Sample Preparation System (Life Technologies, Halle, Belgium), by using the MagMAX™ Nucleic Acid Isolation Kit. The amount of extracted DNA was determined by a Qubit® dsDNA HS Assay Kit (Thermo Fisher Scientific, Vantaa, Finland). 

Each strain-specific DNA was used to verify and validate the accurate qPCR amplification and to ensure the absence of cross-reactivity of the strains. Primers and probes used are indicated in Table 1. 

The PCR reactions were performed on 7500FAST real-time PCR instruments (Applied Biosystems, Waltham (MA), USA) using the FAST protocols and 2X master mixes for SYBR or Taqman chemistries. Briefly, thermocycling consisted of a 20 second 95 °C holding stage followed by 40 cycles of 3 seconds at 95 °C then 30 seconds at primer’s respective annealing temperature. For the *B. lactis* Bi-07 and *B. lactis* Bl-04 assays melt curve analysis was included to verify specificity of amplification.

### 2.4. Statistical Analysis

No power calculation was made for the probiotic strain recovery as it was a secondary end point in the study.

Samples that had no detectable levels of the tested probiotics were given a value of half of their detection limit; Log10 1.98 genomes/g feces for *L. acidophilus* NCFM, Log10 1.99 genomes/g feces for *L. paracasei* Lpc-37, Log10 3.085 genomes/g feces for *B. lactis* Bi-07, and Log10 1.35 genomes/g feces for *B. lactis* Bl-04. Differences in *L. acidophilus* NCFM, *L. paracasei* Lpc-37, *B. lactis* Bi-07, and *B. lactis* Bl-04 levels over time and between treatments were analyzed by Student’s t-test (Microsoft Excel 365, Redmont (WA), USA). As more than 30 observations existed per variable, a normal distribution was assumed. Data on the presence or absence of detectable levels of the test organisms was calculated by Fisher’s exact test (GraphPad Prism 8, La Jolla (CA), USA).

## 3. Results

**Figure 2:**
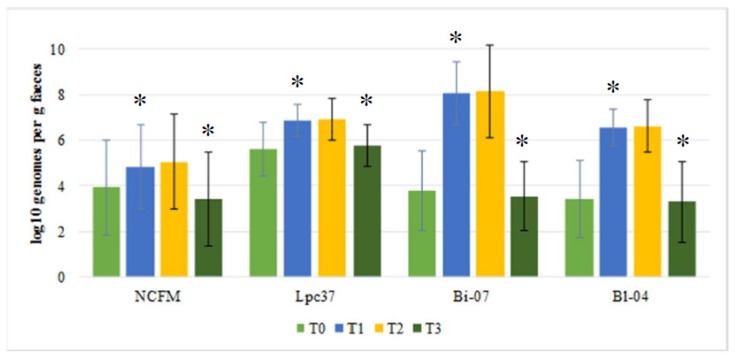
Mean levels (±SE) for Log10 detected genomes per gram feces for the three target strains; *Lactobacillus acidophilus* NCFM, *Lactobacillus paracasei* Lpc-37, *Bifidobacterium lactis* Bi-07, and *B. lactis* Bl-04 in the probiotic group for the four sampling times. Where T0 = baseline, T1 = end of antibiotic treatment, T2 = end of probiotic treatment, and T3 = one week after end of probiotic treatment (washout). * Indicates significant changes from previous time point.

In the probiotic group, there was an increase in detected *L. acidophilus* NCFM, *L. paracasei* Lpc-37, *B. lactis* Bi-07, and *B. lactis* Bl-04 counts over time from baseline (T0) to end of antibiotic treatment (T1). The counts of *L. paracasei* Lpc-37, *B. lactis* Bi-07, and *B. lactis* Bl-04 increased significantly (*p* < 0.0001) (see Figure 2). Moreover, the counts of *L. acidophilus* NCFM increased significantly (*p* = 0.026). No change in counts was observed between end of antibiotic treatment (T1) and end of probiotic treatment (T2) for any of the strains (see Figure 2). However, from end of probiotic treatment (T2) to end of washout (T3), counts decreased significantly for *L. paracasei* Lpc-37, *B. lactis* Bi-07, and *B. lactis* Bl-04 (*p* < 0.0001), and for *L. acidophilus* NCFM (p = 0.0003) and returned to baseline levels, Figure 2. 

**Figure 3:**
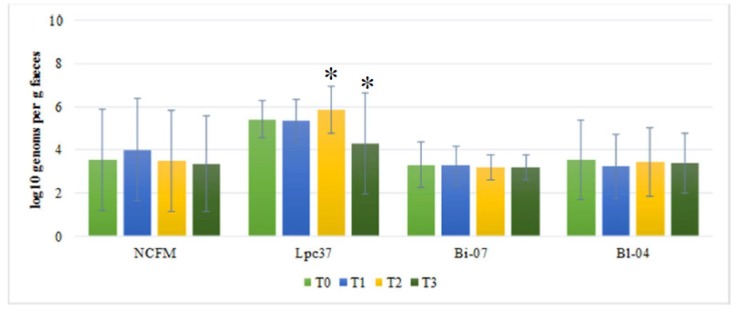
Mean levels (±SE) for Log10 detected genomes per gram feces for the target strains; *Lactobacillus acidophilus* NCFM, *Lactobacillus paracasei* Lpc-37, *Bifidobacterium lactis* Bi-07, and *B. lactis* Bl-04 in the placebo group for the four sampling times. Where T0 = baseline, T1 = end of antibiotic treatment, T2 = end of Placebo treatment, and T3 = one week after end of placebo treatment (washout). * Indicates significant changes from previous time point.

In the placebo group, there was no change in detected for *B. lactis* Bl-04, *B. lactis* Bi-07, and *L. acidophilus* NCFM-like organisms over time from one time point to the next (see Figure 3). However, *L. paracasei* Lpc-37-like organisms increased from end of antibiotic treatment (T1; Log10 5.33) to end of probiotic treatment (T2; Log10 5.84 genomes/g feces; p = 0.021) and decreased after washout (T3; Log10 4.29 genomes/g feces; p = 0.0001) (see Figure 3).

There was no difference between probiotic and placebo groups at baseline (T0) for any of the four strains (see Figure 2 and Figure 3). For *B. lactis* Bi-07, *B. lactis* Bl-04, and *L. paracasei* Lpc-37, significantly higher levels were observed in the probiotic group as compared to placebo group for end of antibiotic treatment (T1; *p* < 0.0001 for all three) and end of probiotic treatment (T2; *p* < 0.0001 for all three). For *L. acidophilus* NCFM, there was a trend (*p* = 0.053) for increased levels in the placebo group during end of antibiotic treatment (T1) and increased (*p* = 0.001) levels in the placebo group at the end of probiotic treatment (T2). Only for *L. paracasei* Lpc-37 there was a difference between placebo and probiotic after washout (T3), with lower levels for the placebo group (*p* = 0.0001) (see Figure 2 and Figure 3).

In the probiotic group, the number of subjects with detectable *L. paracasei* Lpc-37, *B. lactis* Bi-07 and *B. lactis* Bl-04 was a significantly (*p* < 0.0001) increased from baseline (T0) to end of antibiotic treatment (T1) and significantly reduced from end of probiotic supplementation (T2), to after washout (T3; *p* < 0.0001) (see Table 2). For *B. lactis* Bl-04, the number of subjects with detectable levels also reduced significantly (*p* = 0.03) from end of antibiotic treatment (T1) to end of probiotic supplementation (T2), Table 2.

In the placebo group, there was no change in number of subjects with detectable levels of *L. acidophilus* NCFM, *B. lactis* Bl-04 and *B. lactis* Bi-07-like organisms for any of the time points. There was, however, an increase in subjects with detectable *L. paracasei* Lpc-37-like organisms from end of antibiotic treatment (T1) to end of placebo supplementation (T2), there were no changes between other subsequent timepoints, Table 2.

During end of antibiotic treatment (T1) and end of probiotic/placebo supplementation (T2) significantly more subjects had detectable *B. lactis* Bi-07, *B. lactis* Bl-04-like organisms, and *L. paracasei* Lpc-37-like organisms in the probiotic group then in the placebo group (*p* < 0.0001 for both time points and all three target organisms). There was no difference in number of subjects testing positive for *L. acidophilus* NCFM between treatments for any of the time points (see Table 2).

## 4. Discussion

A number of studies have documented the detection of an administered probiotic as it transits the gut, by means of fecal recovery studies. Older studies typically relied upon selective culturing while more recent studies have employed DNA based techniques. The studies that have reported on fecal detection are commonly focusing on a single probiotic strain. Studies that have reported on the fecal recovery of strain combinations have usually focused on strains from different genera, e.g., Bifidobacterium and Lactobacillus [11,12]. Here, we report on the detection of two species from two different genera following their consumption by patients on antibiotic therapy.

Some earlier studies have assessed whether probiotics are detectable in human feces after consumption by healthy volunteers. The strains included in the current study have all been reported to survive gastrointestinal transit and have been detected in feces: *L. acidophilus* NCFM [12,13]; *L. paracasei* Lpc-37 [14]; *B. lactis* Bl-04 [12], and *B. lactis* Bi-07 [15]. Moreover, other probiotic strains have been reported to be detected in feces after consumption, e.g., *Lactobacillus rhamnosus* GG and *B. lactis* Bb-12 [11]. It is also commonly observed that probiotics can no longer be detected approximately 1 week after consumption has seized [11].

A background of organisms like the ones consumed was detected by the used primer sets. This is not uncommon and has been reported in other fecal detection studies, see, e.g., in [11]. However, after consumption, the levels of these organisms markedly and significantly increased. Further, in the present study, a background level was detected for strains like the consumed probiotics. Nevertheless, the levels of the strains were observed to increase significantly and decreased to baseline levels one week after consumption seized. Moreover, with the exception of *L. acidophilus* NCFM, the number of subjects with detectable levels of the strains increased significantly during these periods. The amounts of the probiotic strains consumed were identical: 2.5 × 10^9^ CFU/strain. Nevertheless, the levels after end of the antibiotic treatment (T1) and at the end of the probiotic treatment (T2) were different for the strains, being highest for *B. lactis* Bi-07 (Log10 8.1 genomes/g feces), lowest for *L. acidophilus* NCFM (Log10 5.0 genomes/g feces) and similar for *B. lactis* Bl-04 (Log10 6.6 genomes/g feces) and *L. paracasei* Lpc-37 (Log10 6.8 genomes/g feces). This may indicate that there is a difference in survival between the strains. However, when tested in vitro, such differences were not observed [7]. The low detection of *L. acidophilus* NCFM is difficult to explain, as earlier studies have reported substantially higher fecal recovery levels: 6.6 and 6.7 Log10 genomes/g feces [12,13]. The number of subjects with detectable probiotics was similar for *L. paracasei* Lpc-37, *B. lactis* Bl-04, and *B. lactis* Bi-07, but was less for *L. acidophilus* NCFM; this is in line with earlier observations on fecal recovery of these strains in healthy subjects not consuming antibiotics [9].

The patients were consuming probiotics while on antibiotic therapy. Despite this, the detected levels in the feces were not different between T1 and T2, and neither were the number of subjects with detectable strains different between the two periods; with the exception of *L. paracasei* Lpc-37 which had reduced numbers from T1 to T2. This is in line with earlier reports [16]. This detection happens even though the strains are sensitive to the used antibiotic treatments [17].

## 5. Conclusions

In a four-strain combination of *L. acidophilus* NCFM, *L. paracasei* Lpc-37, *B. lactis* Bi-07, and *B. lactis* Bl-04 can be detected in the feces of patients apparently unaffected by the simultaneous consumption of antibiotics, although at different levels.

## Figures and Tables

**Table 1:** qPCR assays used for bacterial quantification.

Species	Primer Name	Sequence	Reference
*Bifidobacterium animalis* subsp. *lactis* Bl-04	Bl04_for	CTTCCCAGAAGGCCGGGT	[8]
	Bl04_rev	CGAGGCCACGGTGCTCATATAGA	
*Bifidobacterium animalis* subsp. *lactis* Bi-07	Blac_CRins_qF	CGCCGCTGATTGACCTGTT	this manuscript
	Blac_CRins_qP	5FAM-ACGTGACGAATCATGGGCCGAGGGAT-2BHQ	
	Blac_CRins_qR	TGAGATTGATACCCGTGGCG	
*Lactobacillus acidophilus* NCFM	Laci_NCFMMJ_RTfwd	CCACGACCAGATGTAACCAA	[9]
	Laci_NCFM_Rtrev	TTAGAAGATGCCAACGTCGAG	
	Laci_NCFM_probe	5’HEX TAA GCC GAA-ZEN- CAA TGC TGA AAC GAT 3’IABkFQ	
*Lactobacillus paracasei*	F_paca_IS	ACATCAGTGTATTGCTTGTCAGTGAATAC	[10]
	R_paca_IS	CCTGCGGGTACTGAGATGTTTC	
	P_paca_IS	5’ FAM TGCCGCCGGCCAG 3’ IBQ	

**Table 2:** Prevalence of subjects with detectable levels of the studied probiotics; *Lactobacillus acidophilus* NCFM, *Lactobacillus paracasei* Lpc-37, *Bifidobacterium lactis* Bi-07, and *B. lactis* Bl-04. Where T0 = baseline, T1 = end of antibiotic treatment, T2 = end of probiotic or placebo treatment, and T3 = one week after end of probiotic or placebo treatment (washout).

Intervention Group	Probiotic (n = 46)				Placebo (n = 50)			
	NCFM	Lpc-37	Bi-07	Bl-04	NCFM	Lpc-37	Bi-07	Bl-04
**T0**	4	20	7	5	7	18	2	2
**T1**	7	42^a^**	43^a^**	44^a^**	9	15^a^	2 ^a^	4^a^
**T2**	10	42^a^	40^a^	37^a^*	16	25^a^*	1 ^a^	5^a^
**T3**	3	23**	3**	4**	6	24	1	4

* *p* < 0.05, ** *p* < 0.0001 compared to previous time point; Fisher’s exact test. ^a^ Significantly different (*p* < 0.0001) between treatment groups (i.e., probiotic and placebo) for same time point and target organism.

## Abbreviations

AAD	Antibiotic associated diarrhea
CFU	Colony forming units
SE	Standard error

## References

[1] Wiström J., Norrby S.R., Myhre E.B., Eriksson S., Granström G., Lagergren L., Englund G., Nord C.E., Svenungsson B. (2001). Frequency of antibiotic-associated diarrhoea in 2462 antibiotic-treated hospitalized patients: A prospective study. J. Antimicrob. Chemother..

[2] McFarland L.V. (1998). Epidemiology, risk factors and treatments for antibiotic-associated diarrhea. Dig. Dis..

[3] McFarland L.V. (2006). Meta-analysis of probiotics for the prevention of antibiotic associated diarrhea and the treatment of Clostridium difficile disease. Am. J. Gastroenterol..

[4] Haran J.P., Hayward G., Skinner S., Merritt C., Hoaglin D.C., Hibberd P.L., Lu S., Boyer E.W. (2014). Factors influencing the development of antibiotic associated diarrhea in ED patients discharged home: Risk of administering IV antibiotics. Am. J. Emerg. Med..

[5] Blaabjerg S., Artzi D.M., Aabenhus R. (2017). Probiotics for the Prevention of Antibiotic-Associated Diarrhea in Outpatients-A Systematic Review and Meta-Analysis. Antibiotics.

[6] Ouwehand A.C., DongLian C., Weijian X., Stewart M., Ni J., Stewart T., Miller L.E. (2014). Probiotics reduce symptoms of antibiotic use in a hospital setting: A randomized dose response study. Vaccine.

[7] Forssten S.D., Ouwehand A.C. (2017). Simulating colonic survival of probiotics in single-strain products compared to multi-strain products. Microb. Ecol. Health Dis..

[8] Hansen S.J.Z., Morovic W., DeMeules M., Stahl B., Sindelar C.W. (2018). Absolute Enumeration of Probiotic Strains *Lactobacillus acidophilus* NCFM® and *Bifidobacterium animalis* subsp. *lactis* Bl-04® via Chip-Based Digital PCR. Front. Microbiol..

[9] Airaksinen K., Yeung N., Lyra A., Lahtinen S.J., Huttunen T., Shanahan F., Ouwehand A.C. (2019). The effect of a probiotic blend on gastrointestinal symptoms in constipated patients: A double blind, randomised, placebo controlled 2-week trial. Benef. Microbes.

[10] Haarman M., Knol J. (2006). Quantitative real-time PCR analysis of fecal Lactobacillus species in infants receiving a prebiotic infant formula. Appl. Environ. Microbiol..

[11] Poutsiaka D.D., Mahoney I.J., McDermott L.A., Stern L.L., Thorpe C.M., Kane A.V., Baez-Giangreco C., McKinney J., Davidson L.E., Leyva R. (2017). Selective method for identification and quantification of *Bifidobacterium animalis* subspecies lactis BB-12 (BB-12) from the gastrointestinal tract of healthy volunteers ingesting a combination probiotic of BB-12 and *Lactobacillus rhamnosus* GG. J. Appl. Microbiol..

[12] Ouwehand A.C., Nermes M., Collado M.C., Rautonen N., Salminen S., Isolauri E. (2009). Specific probiotics alleviate allergic rhinitis during the birch pollen season. World J. Gastroenterol..

[13] Ouwehand A.C., ten Bruggencate S.J., Schonewille A.J., Alhoniemi E., Forssten S.D., Bovee-Oudenhoven I.M. (2014). Lactobacillus acidophilus supplementation in human subjects and their resistance to enterotoxigenic Escherichia coli infection. Br. J. Nutr..

[14] Hemalatha R., Ouwehand A.C., Forssten S.D., Babu Geddan J.J., Sriswan Mamidi R., Bhaskar V., Radhakrishna K.V. (2014). A Community-based Randomized Double Blind Controlled Trial of Lactobacillus paracasei and Bifidobacterium lactis on Reducing Risk for Diarrhea and Fever in Preschool Children in an Urban Slum in India. Eur. J. Nutr. Food Saf..

[15] Bettler J., Mitchell D.K., Kullen M.J. (2006). Administration of Bifidobacterium lactis with fructo-oligosaccharides to toddlers is safe and results in transient colonization. Int. J. Probiotics Prebiotics.

[16] Forssten S., Evans M., Wilson D., Ouwehand A.C. (2014). Influence of a probiotic mixture on antibiotic induced microbiota disturbances. World J. Gastroenterol..

[17] Morovic W., Roper J.M., Smith A.B., Mukerji P., Stahl B., Rae J.C., Ouwehand A.C. (2017). Safety evaluation of HOWARU Restore® (*Lactobacillus acidophilus* NCFM, *Lactobacillus paracasei* Lpc-37, *Bifidobacterium animalis* subsp. *lactis* Bl-04 and *B. lactis* Bi-07) for antibiotic resistance, genomic risk factors, and acute toxicity. Food Chem. Toxicol..

